# Structural, energetic, and dynamic insights into the abnormal xylene separation behavior of hierarchical porous crystal

**DOI:** 10.1038/srep11537

**Published:** 2015-06-26

**Authors:** Jiao-Min Lin, Chun-Ting He, Pei-Qin Liao, Rui-Biao Lin, Jie-Peng Zhang

**Affiliations:** 1 MOE Key Laboratory of Bioinorganic and Synthetic Chemistry, School of Chemistry and Chemical Engineering, Sun Yat-Sen University, Guangzhou 510275, P.R. China

## Abstract

Separation of highly similar molecules and understanding the underlying mechanism are of paramount theoretical and practical importance, but visualization of the host-guest structure, energy, or dynamism is very difficult and many details have been overlooked. Here, we report a new porous coordination polymer featuring hierarchical porosity and delicate flexibility, in which the three structural isomers of xylene (also similar disubstituted benzene derivatives) can be efficiently separated with an elution sequence inversed with those for conventional mechanisms. More importantly, the separation mechanism is comprehensively and quantitatively visualized by single-crystal X-ray crystallography coupled with multiple computational simulation methods, in which the small apertures not only fit best the smallest *para*-isomer like molecular sieves, but also show seemingly trivial yet crucial structural alterations to distinguish the *meta*- and *ortho*-isomers via a gating mechanism, while the large channels allow fast guest diffusion and enable the structural/energetic effects to be accumulated in the macroscopic level.

Xylene and many other disubstituted benzene derivatives are generally produced as mixtures of the *ortho*- (*o*-), *meta*- (*m*-) and *para*- (*p*-) isomers. Separation, detection and identification of such isomers with very similar chemical and physical properties are of great importance and challenge in industry and environmental sciences^1^,^2^,^3^,^4^,^5^,^6^,^7^,^8^,^9^,^10^,^11^. Compared with conventional distillation or crystallization methods, separating mixtures by differential interaction with solid surface could be more energy saving^6^. The isomer possessing higher polarity and polarizability (*o*-, *m*-, and *p*-xylenes, denoted as oX, mX, and pX, respectively, follow oX > mX ≈ pX as reflected by their boiling points) interacts stronger with the surface to give stronger adsorption or longer retention time. However, because the physical properties of the oX, mX, and pX are extremely similar, complete separation can be hardly achieved by the differential interaction mechanism, even when porous solids with high surface areas are used to enhance the host-guest interaction^12^,^13^,^14^,^15^. Much higher separation selectivity can be obtained by using porous materials with strictly defined pore sizes, such as zeolites or molecular sieves, which only adsorb/retain and effectively isolate the smallest one (pX) from the three isomers. Besides the difficulty for separating oX and mX^1^,^4^,^16^, the very small pore sizes of zeolites/molecular sieves also limit the guest diffusion rate and separation performance.

Porous coordination polymers (PCPs) or metal-organic frameworks are new generation adsorbents combining advantages of versatile/tunable framework^17^, pore^18^, and surface structures^19^, large surface areas^20^, as well as notable framework flexibilities^21^,^22^,^23^,^24^,^25^. Besides the general differential interaction^12^,^13^,^14^ and molecular sieving mechanisms^16^, PCPs may undergo significant structural alteration to allow accommodation of a specific isomer (generally the smallest one, pX)^26^,^27^, achieving similar separation performance as for molecular sieves without the need of accurate adjustment of the pore size. Less selective but complete separation may be possible by using PCPs with special pore sizes/shapes which force the three isomers to pack differently to achieve different adsorption capacities, although reported examples so far can only separate one isomer among the three^28^,^29^,^30^,^31^,^32^. In principle, the synergetic effect of two or more separation mechanisms might be realized in a single PCP crystal to obtain optimized separation performances, for which understanding the structural, energetic, and dynamic mechanism of the host-guest system is fundamental.

Owning to the long-range ordered crystal structures of PCPs, the host-guest interacting structures may be directly and straightforwardly visualized at the molecular level by diffraction techniques, especially using single-crystal samples^26^,^27^,^33^,^34^,^35^,^36^,^37^,^38^,^39^,^40^. However, because the sample crystallinity tends to degrade during the processes of adsorbent activation and/or adsorption, and the guest molecules generally show large dynamic motion, determination of the host-guest structures, especially the location and orientation of guests is always very difficult. On the other hand, as a method for long-range averaged structural information at the thermodynamic equilibrium state, crystallography may qualitatively judges the relative strengths of host-guest interactions (i.e., cannot quantitatively determine the host-guest interaction energies) and can hardly reveal the dynamic or kinetic information of the guest molecules. In this regard, computational simulation can serve as an important supplementary for studying the energetic and kinetic aspects of the host-guest system^41^,^42^,^43^,^44^,^45^,^46^,^47^.

Herein, we report a unique xylene separation mechanism observed for a new PCP featuring hierarchical and flexible pore structure, in which the host-guest structures, energies, and dynamisms are comprehensively and quantitatively visualized by combined single-crystal X-ray diffraction (SCXRD) and computational simulation studies.

## Results

### Preparation and characterization of the porous crystal

**Figure 1:**
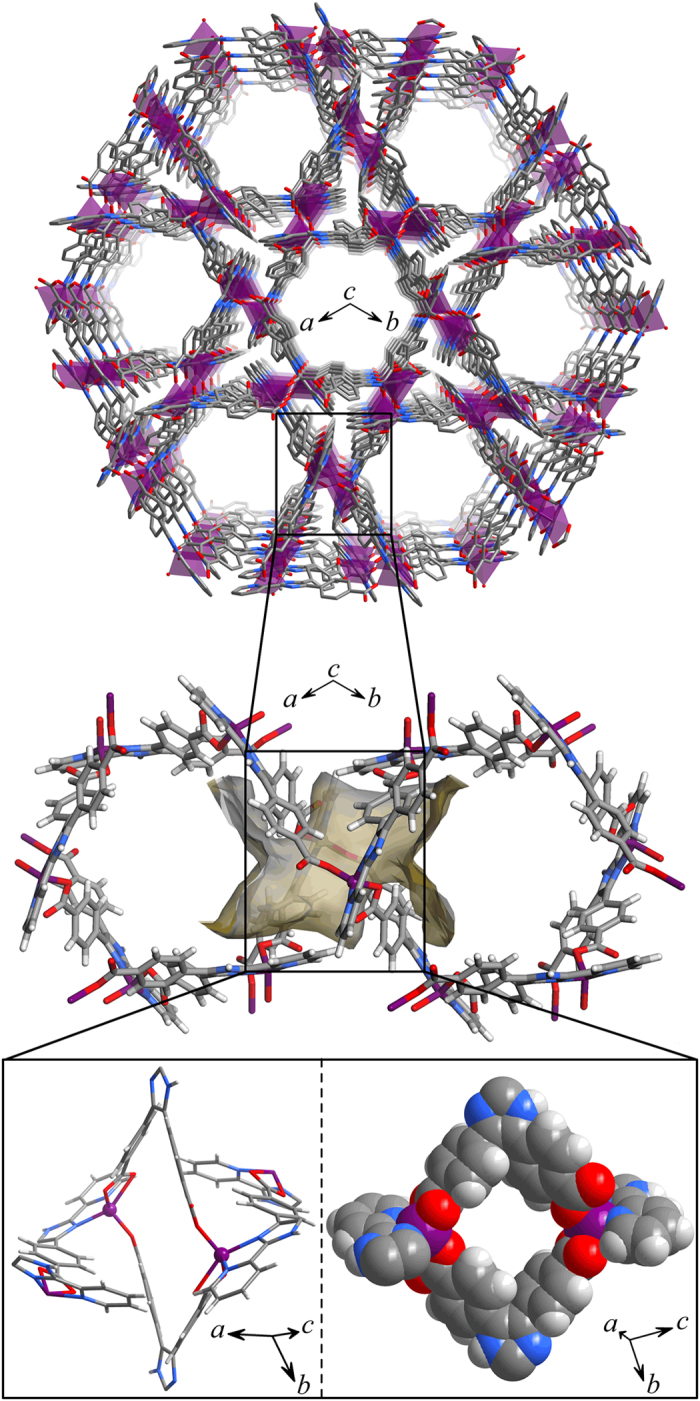
The 3D coordination network and local coordination/pore structures. The quadrilateral aperture (defined by the yellow sticks) is shown in the space-filling mode to highlight its effective aperture size/shape.

Solvothermal reaction of 4,4’-(2-(pyridin-2-yl)-1*H*-imidazole-4,5-diyl)dibenzoic acid (H_3_pidba) and ZnCl_2_ in *N*,*N*-dimethylformamide (DMF) gave light yellow block crystals of a new metal carboxylate framework [Zn(Hpidba)]∙2.6DMF∙H_2_O (MCF-50, **1∙g**) in high yield. SCXRD revealed that **1∙g** processes a high-symmetry (space group *R*-3) three-dimensional (3D) non-interpenetrated coordination network (Table S1 and Figures S1-S2) and a hierarchical pore system, in which large 1D hexagonal channels (effective diameter 8.2–8.7 Å, van der Waals radii of atoms considered) running along the *c*-axis are interconnected through small quadrilateral apertures (effective height and width of 5.6 and 6.8 Å along the imidazole· · ·imidazole and Zn· · ·Zn diagonals, respectively) running along the *a*- and *b*-axes (Fig. 1).

Thermogravimetry analysis and variable-temperature powder X-ray diffraction showed that **1∙g** can be readily activated by exchanging the guest with CH_3_OH and then heated at 50 °C, which gives guest-free **1** stable up to 350 °C (Figures S3–S5). The framework stability of **1** was further confirmed by successful measurement of its single-crystal structure (although it tends to crack during activation), which possesses a slightly expanded unit-cell volume (+3.5%). Comparison of the crystal structures of **1∙g** and **1** showed that the conformation change of the organic ligand is responsible for the framework flexibility. The 3D intersecting channel occupies 48.4% volume of **1**, corresponding to a crystallographic pore volume of 0.513 cm^3^ g^−1^ (crystal density 0.944 g cm^−3^).

The porosity of **1** was evaluated by N_2_ and CO_2_ gas sorption at 77 K and 195 K, respectively. The N_2_ isotherm exhibits a two-step character with an obvious hysteresis at *P*/*P*_0_ = 0.24–0.56 (Figure S6). The saturated uptakes of the two steps are 351 and 433 cm^3^ g^−1^, corresponding to pore volumes of 0.548 and 0.677 cm^3^ g^−1^, respectively. Considering that the theoretical value empirically calculated from the crystal structure of **1** is 0.513 cm^3^ g^−1^, the second isotherm step should be attributed to a significant expansion of the framework. The BET and Langmuir surface areas were calculated to be 1319 and 1512 m^2^ g^−1^ by using the first step of the N_2_ isotherm, respectively. On the other hand, the CO_2_ sorption isotherm exhibits the normal type-I character with a saturated adsorption capacity of about 308 cm^3^ g^−1^ (Figure S6), giving a pore volume of 0.550 cm^3^ g^−1^, which is coincided with that calculated from the first plateau of the N_2_ isotherm. The relatively high pressure for starting the second N_2_ adsorption step and one-step CO_2_ adsorption isotherm indicate that **1** is flexible but significant structural alteration is not as easy as other typical flexible PCPs^48^.

### Separation of structural isomers of disubstituted benzene derivatives

**Figure 2:**
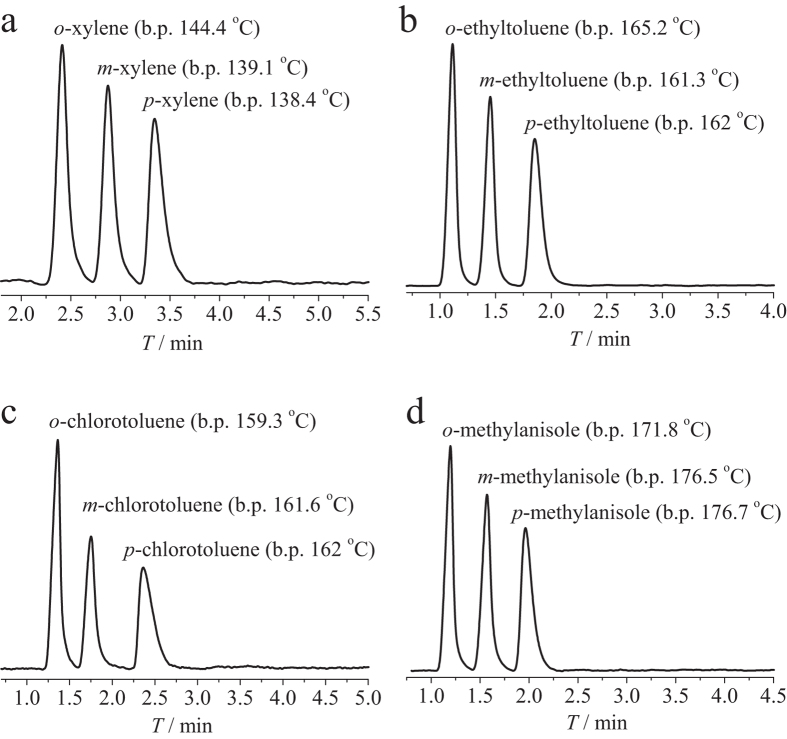
Chromatograms of GC separation of structural isomers of disubsituted benzene derivatives. **a**. Separation of xylene isomers (1.2 μg each isomer) using a temperature program of 180 to 200 °C with a rate of 5 °C min^−1^, under a N_2_ flow rate of 14 mL min^−1^. **b**. Separation of ethyltoluene isomers (2 μg each isomer) at 230 °C under a N_2_ flow rate of 14 mL min^−1^. **c**. Separation of chlorotoluene isomers (4 μg each isomer) at 215 °C under a N_2_ flow rate of 14 mL min^−1^. **d**. Separation of methylanisole isomers (2 μg each isomer) at 230 °C under a N_2_ flow rate of 14 mL min^−1^.

Considering the hierarchical pore structure and good thermal stability, **1** may be suitable as a stationary material for gas chromatography (GC) separation of highly similar molecules such as isomers of xylene and similar disubstituted benzene derivatives. A capillary column with microcrystalline **1** coated on its inner surface was fabricated by a dynamic coating method (Figure S7)^8^. As shown in Fig. 2a and Table S2, the three xylene isomers are well separated from each other with good precision for retention time, half peak width, and peak area. It is noteworthy that the column can efficiently separate mX and pX with large resolutions of 1.54–1.65 (Figure S8), which are higher than for similar materials such as MIL-101 (0.9–1.0)^8^. More interestingly, the GC elution times follow oX < mX < pX, an order that has not been reported for other materials. For common stationary phases^8^,^49^,^50^, the elution times usually follow pX < mX < oX, being the same as the orders of their boiling points and polarities (dipole moment and polarizability, 0 D and 13.7 cm^3^ for pX, 0.36 D and 14.2 cm^3^ for mX, and 0.44 D and 14.9 cm^3^ for oX, respectively). Comparison of the molecular sizes (Figure S9) and the observed retention times shows that **1** tends to retain the smaller molecules, which is similar to that of molecular sieving effect. However, molecular sieves can only divide the mixture into two groups (smaller/larger than the aperture size), indicating that there should be a distinctive separation mechanism for **1**.

To further verify the size/shape selectivity of **1**, we tested its separation performance for other disubstituted benzene derivatives including ethyltoluene, chlorotoluene, and methylanisole, whose separation is also challenging for most commercial capillary columns such as HP-5MS (Figure S10). As expected, the *o*-, *m*-, and *p*-isomers of these disubstituted benzene derivatives also have the same elution sequences as for the xylene isomers (Fig. 2). It is worth pointing out that the boiling point of *o*-ethyltoluene is also obviously higher than that of the *m*- and *p*-isomers. Although the boiling points of the other tested guests are coincided with their elution times, their separation efficiencies, including selectivities and resolutions, are obviously higher than those based solely on the differences of their boiling points and polarities (Figures S8 and S10-S11). These results confirm that **1** has size/shape selectivity for the *o*-, *m*- and *p*-isomers, rather than the common polarity selectivity. The adsorption enthalpies were calculated by the van’t Hoff equation based on the retention factors at different temperatures (Table S2 and Figure S12), which confirmed that **1** has the highest affinities for the *p*-isomers and lowest for the *o*-isomers.

### Single-crystal X-ray diffraction

To directly visualize the different host-guest interactions and thus understand the separation mechanism, we tried to measure the single-crystal structures of **1** loaded with xylene isomers. Because the single crystals of **1** always crack when they directly contact with xylene vapor or liquid, the xylene-loaded single crystals were obtained through a guest-exchange approach, in which as-synthesized single crystal of **1∙g** was immersed in xylene/DMF mixture with gradually increased xylene concentration and finally in pure xylene at 70 °C to get the completely xylene-exchanged sample (denoted as **1∙oX**, **1∙mX**, and **1∙pX** for the crystals loaded with oX, mX, and pX, respectively).

**Figure 3:**
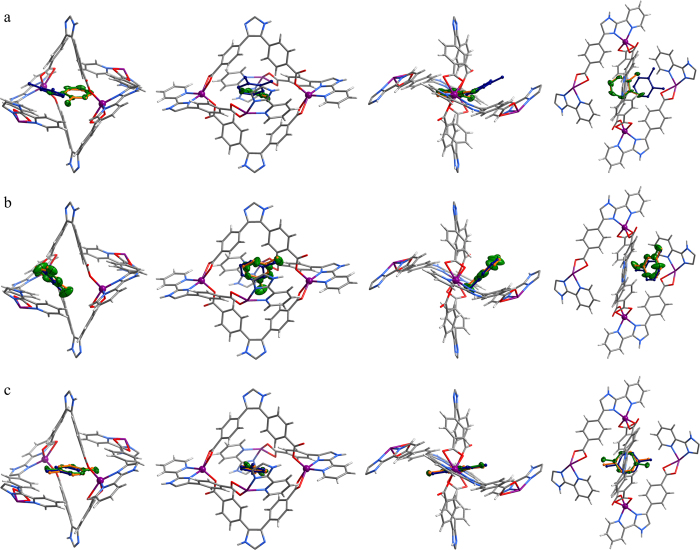
Experimental and simulated host-guest structures. **a**–**c**. Perspective views of local structures of the preferential adsorption site (drawn in stick mode; Zn purple, C dark gray, H light gray, N blue, O red) loaded with oX, mX and pX (hydrogen atoms are omitted for clarity) observed by single-crystal X-ray diffraction (Ortep plot in green, probability drawn at 30%), GCMC simulation (ball-and-stick mode in dark blue) and GCMC-PDFT calculation (ball-and-stick mode in orange). From left to right, the structures are projected along a common direction (same as that for Fig. 1), perpendicular to the aperture plane, parallel to the Zn· · ·Zn diagonal, and parallel to the imidazole· · ·imidazole diagonal, respectively.

The unit-cell volumes of **1∙oX** and **1∙mX** are very similar with that of **1∙g**, while that of **1∙pX** is similar with that of **1** (Table S1), indicating that the host framework can distort differently toward interaction with different guests. Structural analyses revealed that the oX, mX, and pX molecules locate quite differently in **1**. In the large 1D channel, the xylene molecules show serious disorder, indicating weak host-guest interactions and large guest mobility, which indicate that the large channel mainly serves as the guest transportation path rather than the primary adsorption site. Inside and/or very close to the small aperture, the xylene molecules can be anisotropically refined without any restriction, confirming that the small aperture is the primary adsorption site for GC separation (Fig. 3 and S13–S15). The very different thermal parameters of oX, mX, and pX indicate that the host-guest binding strength follow **1∙pX** > **1∙oX** > **1∙mX**, which is not completely consistent with the trend of adsorption enthalpies observed in the GC separation. Therefore, the energy consumptions of the structure alterations of the host framework should be also taken into account.

The oX molecule completely inserts into the aperture, with its phenyl ring and methyl groups residing at two opposite sides of the aperture and its molecular plane parallel with the Zn· · ·Zn diagonal. Considering the effective sizes and shapes of oX (3.83 × 7.27 Å^2^, Figure S9) and the aperture of guest-free **1** (5.6 × 6.8 Å^2^), the guest molecule obviously adopts an orientation that minimizes steric hindrance and host-framework distortion. On the other hand, one methyl group of mX inserts a little into the quadrilateral aperture, while the other part of the molecule lies on the pore wall outside the aperture. Similar with oX, pX also completely inserts into the quadrilateral aperture, with its molecular center exactly overlapping with that of the aperture.

Obviously, all three xylene isomers try to insert their smallest cross sections into the aperture. In this context, mX with a smallest cross-section size almost identical with that of oX should completely enter the aperture, but this is not observed in the crystal structure. From the observed host-guest structure, it can be seen that the mX molecule already adopted the best orientation for insertion (see blue arrows in Figure S9). Nevertheless, the head part of mX just stopped at the aperture entrance, implying that its tail part is too large (steric hindrance). If mX adopts the position of pX or oX, at least one of its two methyl groups would form unacceptably close contacts with the pore wall atoms (Figure S16). Another explanation for the observed host-guest structure is that the tail part of mX tends to interact with the outside pore wall. In fact, the tail part of mX formed multiple weak interactions with the pore wall atoms, including a C–H· · ·O hydrogen bond with the carboxylate oxygen (C· · ·O 3.96(2) Å) and π–π interaction with the pyridyl ring (shortest C· · ·C 3.55(1) Å, C· · ·N 3.78(1) Å) (Figure S15). The very different host-guest crystal structures highlight that the aperture of **1** can effectively distinguish both the size and shape of xylene isomers.

In **1∙oX**, the aperture width expands significantly (+0.36 Å) compared with that of guest-free **1** (Table 1), indicating large steric hindrance or repulsion effects between the host framework and guest molecule, as well as large structural transformation energy for the adsorption. For mX, the aperture width expands with a smaller extend (+0.32 Å), indicating that less structural transformation energy is required for mX as compared with oX. As the molecular width of pX is shorter than the effective width of the aperture, the host-guest attractive interactions slightly reduce the aperture width (−0.09 Å), meaning lowest structural transformation energy in the adsorption process. Further, we treated the crystal with a mixture of equimolar oX, mX, and pX and then determined its single-crystal structure (denoted as **1∙ompX**, Table S1), in which the quadrilateral apertures are indeed occupied only by pX molecules. These observations exemplify that the porous crystal **1** can fit best and have strongest adsorption (highest enthalpy) for pX among the three xylene isomers, and indicate that the abnormal retention trend between oX and mX is very likely resulted from the combination effects of host-guest fitting and structural transformation of the host.

### Computational simulation

To quantitatively understand the structure-energy relationship, the xylene adsorption mechanisms were further investigated by computational simulations. We first used grand canonical Monte Carlo (GCMC) simulation, which allows the guest molecule to freely rotate and translate while treats both the host framework and guest molecule as rigid bodies, to obtain the primary adsorption sites and corresponding host-guest structures. In the most energetically favored simulation results, mX and pX were found at positions close to those determined by SCXRD (Fig. 3b,c and S14b). However, oX resides at the entrance instead of completely inserted into the aperture like that observed by SCXRD (Fig. 3a). These observations indicate that the original aperture of **1** is not large enough for accommodation of oX and mX, and the flexibility of host-framework should be taken into consideration to obtain reliable host-guest structures and energies. We then carried out periodic density functional theory (PDFT) calculation, which allows the whole host-guest structure to change, to further optimize the host-guest structures obtained by GCMC simulation. As expected, oX completely inserts into the aperture in this case, while mX and pX barely change their positions during the PDFT optimization (Fig. 3 and Figure S14c).

From the host-guest structures and energies obtained by combined GCMC-PDFT calculations (Table 1), the host-guest fitting energies (*ΔE*_fitting_)^51^ of the final host-guest structures were calculated as pX (−61.95 kJ mol^−1^) > oX (−58.48 kJ mol^−1^) > mX (−58.27 kJ mol^−1^). However, to adsorb the guest molecules, the host framework undergoes different structural distortions from the guest-free form, in which the small aperture expands 0.09 Å and 0.04 Å for oX and mX, respectively, and shrinks 0.03 Å for pX (Table 1). These results are consistent with those observed by SCXRD. As a result, the framework distortions consume energies (*ΔE*_trans_) of 3.32, 0.88 and 2.21 kJ mol^−1^ for oX, mX, and pX, respectively. Taking the host-guest fitting and host-framework distortion energies both into consideration, the total adsorption heats (*ΔE*_ads_) can be calculated as –55.16, –57.39 and –59.74 kJ mol^−1^ for oX, mX, and pX, respectively, being consistent with the trend observed by GC (Table 1).

**Figure 4:**
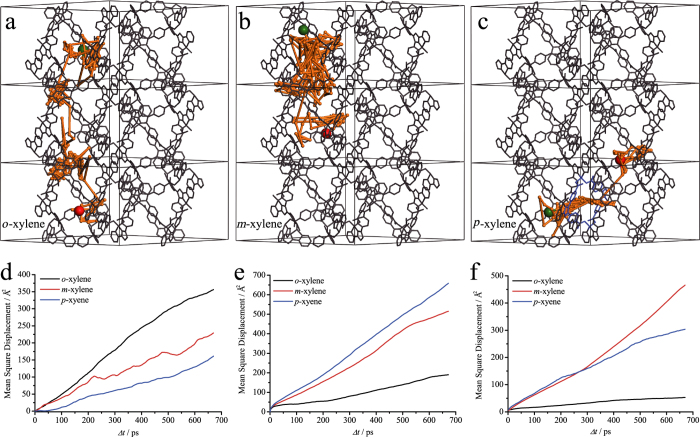
Diffusion behaviors of xylene isomers in the hierarchical porous crystal. **a**–**c**. Moving trajectories (orange sticks, with the starting and ending points highlighted in green and red spheres) of xylene isomers in **1** within 450 ps. The host frameworks are shown in the stick mode in gray (hydrogen atoms are omitted for clarity) with the aperture passed by the guest highlighted in blue. **d**–**f**. The self-diffusion rates of xylene isomers in **1** (pristine), **1a** (pX-blocked), and **1b** (rigid).

To visualize the overall adsorption and diffusion behaviors of the xylene isomers in the hierarchical pore system of **1**, we carried out molecular dynamics (MD) simulations with the same temperature (453 K) for the GC experiments. As shown by the obtained guest moving trajectories (Fig. 4a–c), all three isomer molecules mainly hop around the entrance of the small aperture and travel fast along the large 1D channel, confirming that the small aperture is the primary adsorption site and the large 1D channel is the transportation path for the fast guest diffusion. Interestingly, the pX molecule can occasionally past through the small aperture and diffuse to the adjacent 1D channels. In contrast, oX and mX molecules only appear in the original 1D channel. These phenomena show that although pX and oX can both completely insert into the small aperture, only the one with the smallest cross-section area can easily pass through, which can be attributed to their very different steric hindrances and structure transformation energies of the host framework. It should be noted that, without the MD simulation, one cannot judge the difficulty for mX to pass through the small aperture, because the crystal structure and GCMC/PDFT simulation cannot capture the high-energy transient states.

According to the time-dependent mean square displacement of the guest molecules (Fig. 4d), the mobility of xylene isomers in **1** clearly follows oX > mX > pX, which is consistent with their differences of crystal and GCMC-PDFT structures/energies, as well as the experimental GC elution sequence. Based on the Einstein’s equation, the self-diffusion coefficients of oX, mX, and pX can be fitted as 9.53(5) × 10^−10^, 5.25(2) × 10^−10^, and 3.81(2) × 10^−10^ m^2^/s, respectively, which are similar with other PCPs with channel diameters of ca. 10 Å^42^,^44^. The importance of the small and flexible apertures of **1** for the abnormal xylene separation behavior can be also demonstrated by MD simulations for two hypothetical materials derived from **1**, in which pX molecules were fixed into the small apertures as observed by the crystal structure (**1a**) or the host framework was treated as a rigid body (**1b**). Without the small aperture, the self-diffusion coefficient of xylene isomers in **1a** follows oX < mX < pX, which is conversed with that for **1** but the same as those for conventional stationary materials based on differential interaction mechanism (Fig. 4e). Without framework flexibility, the self-diffusion coefficient of xylene isomers in **1b** follows oX < pX < mX, which is also different with that for **1** (Fig. 4f).

## Discussion

In summary, a new PCP with large 1D channels interconnected by flexible and small apertures has been synthesized. Using as the stationary phase of a GC capillary column, high-resolution and fast separation performances can be realized for the highly similar *o*-, *m*-, and *p*-isomers of not only xylene but also other disubstituted benzene derivatives including ethyltoluene, chlorotoluene, and methylanisole. Interestingly, all structural isomers of the tested disubstituted benzene derivatives follow a unique elution sequence that different with those for the common separation mechanisms based on differential host-guest interaction and molecular sieving. SCXRD and computational simulations revealed that the flexible and small aperture is the preferential adsorption site, in which the size/shape fitting between the host and guest and the structural distortion of the host framework codetermine the final adsorption enthalpies. The linear *p*-isomer with the smallest cross section area fits best the small aperture, so that both host-guest binding and host distortion favor this guest, giving strongest retention effect similar with that of molecular sieves. For the *o*- and *m*-isomers with larger cross section areas, the situation is complicated, in which much better host-guest fitting and larger host distortion are observed for the former. Consequently, their combination energy effects have to be calculated from the combined GCMC simulation and PDFT optimization, which satisfactorily explain the abnormal selectivity for mX over oX. Finally, the guest diffusion behaviors of the isomers are visualized by MD simulations, which demonstrate that the differences of the local host-guest interplays localized at the small aperture are accumulated by fast diffusion through the large 1D channel. These results may enlighten the design, characterization, and optimization of novel porous materials for separation of highly similar molecules.

## Methods

### Materials

All commercially available reagents and solvents were used as received without further purification. The ligand H_3_pidba was synthesized according to the literature^52^.

### Measurements

Elemental analyses (C, H, and N) were performed with a Vario EL elemental analyzer. Powder X-ray diffraction data were collected on a Bruker D8 ADVANCE X-ray powder diffractometer (Cu Kα). Thermogravimety analyses were performed on a TA Q50 instrument with a ramp rate of 5.0 °C min^−1^ under a nitrogen atmosphere. Scanning electron microscopy (SEM) images were recorded on a Quanta 400 field-emitted SEM device. Sorption isotherms were measured on an automatic volumetric sorption apparatus (BELSORP-max, Bel Japan). Before sorption experiments, the as-synthesized crystalline sample was soaked in methanol for one week, and then placed in the sample tube and dried under high vacuum at 100 °C for 12 h. GC separation experiments for the capillary column coated with **1** were performed on an Agilent 7890A system equipped with a thermal conductivity detector. The data acquisition and processing were controlled by the ChemStation software. Before GC injection, all analytes were first diluted in methanol.

### Synthesis of [Zn(Hpidba)]∙2.6DMF∙H_2_O (MCF-50, 1∙g)

A mixture of ZnCl_2_ (0.027 g, 0.2 mmol), H_3_pidba (0.077 g, 0.2 mmol), and DMF (3 mL) was sealed in a 10-mL Teflon-lined stainless steel container and kept at 120 °C for 3 days, followed by cooling to room temperature at a rate of 5 °C/h. Light yellow block crystals were obtained with the yield of 69% based of Zn. EA calc. (%) for [Zn(Hpidba)]∙2.6DMF∙H_2_O (ZnO_7.6_N_5.6_C_29.8_H_33.2_): C, 54.49; H, 5.09; N, 11.94; found: C, 54.79; H, 4.87; N, 12.24.

### X-ray crystallography

All diffraction data were collected on a Rigaku XtaLAB P300DS single-crystal diffractometer with graphite monochromated Cu Kα radiation (λ = 1.54178 Å). Single crystal specimens were sealed in glass capillaries either in vacuum or with corresponding pure xylene, respectively. Absorption corrections were applied by using the multiscan program REQAB^53^. The structures were solved by the direct method and refined with the full-matrix least-squares technique on *F*^2^ by the *SHELXTL* package^54^. All non-hydrogen atoms of the host frameworks, as well as xylene molecules in the quadrilateral apertures, were refined anisotropically. Hydrogen atoms were placed geometrically. The *PLATON SQUEEZE* treatment was applied to all guest molecules in as-synthesized **1∙g** because the guest solvent molecules are extremely disordered and cannot be modeled. A fraction of xylene in the large 1D channel is also extremely disordered and cannot be modeled, resulting in solvent accessible voids.

### Computational simulation

All simulations/calculations were performed by the Materials Studio 5.0 package. The preferred sorption locations were searched through GCMC simulations by using the loading task in the Sorption module. The simulation box consisted of one unit cell and the Metropolis method based on the universal forcefield (UFF) was used. Both the framework and the xylene molecules were regarded as rigid body and the QEq partial charges and ESP charges were employed to the atoms of the framework and guest molecules, respectively. The cutoff radius was chosen as 18.5 Å for the LJ potential, and the maximum loading steps and the equilibration steps were both 5 × 10^6^ following with 1 × 10^7^ production steps. All energies were calculated by the PDFT method by the Dmol^3^ module. Before the energy calculations, full geometry optimizations with fixed cell parameters were performed to the host-guest systems based on the adsorption conformations from GCMC simulation. The widely used generalized gradient approximation (GGA) with the Perdew-Burke-Ernzerhof (PBE) functional and the double numerical plus d-functions (DND) basis set as well as the DFT Semi-core Pseudopots (DSPP) were used. The energy, gradient and displacement convergence criterions were set as 2 × 10^−5^ Ha, 4 × 10^−3^ Å and 5 × 10^−3^ Å, respectively. The energy balance is expressed by the following equations^51^:


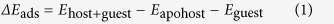



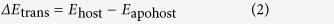



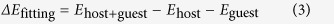


Where *ΔE*_ads_ is the calculated heat of adsorption, *ΔE*_trans_ is the energy change of the host framework after adsorption of guest, *ΔE*_fitting_ is the interaction energy between the guest and the final host framework, and *E*_host+guest_, *E*_guest_, *E*_apohost_ and *E*_host_ are the energies of the guest-adsorbed structure, the guest, the guest-free host framework, and the transformed host framework after adsorption, respectively.

The diffusion behaviors were simulated by MD. The initial configurations for the MD simulations were produced by the GCMC loading results followed with global geometry optimizations through the universal forcefield with the same partials charges as mentioned above. All of the MD processes adopted the canonical ensemble with constant volume/temperature (NVT) using Nose thermostat and random initial velocities. The time step was 1.0 fs with relaxation time 2.0 ps and total simulation time 4 ns. The electrostatic interactions and the van der Waals interactions were evaluated by the Ewald summation method, while all the Buffer widths were set as 0.5 Å. The first 2 ns were used as equilibrium and the following 2 ns were adopted for statistical analysis such as for mean square displacements.

## Additional Information

**How to cite this article**: Lin, J.-M. *et al*. Structural, energetic, and dynamic insights into the abnormal xylene separation behavior of hierarchical porous crystal. *Sci. Rep*. **5**, 11537; doi: 10.1038/srep11537 (2015).

## Supplementary Material

Supplementary Information

## Figures and Tables

**Table 1:** Structure and energy differences for separation of xylene isomers.

Guest	Null	oX	mX	pX
Simulated/experimental aperture size (Å)^a^	7.647/7.449(1)	7.736/7.811(3)	7.687/7.771(2)	7.613/7.363(2)
Simulated/experimental aperture size variation (Å)^a^	NA	0.09/0.36	0.04/0.32	−0.03/−0.09
*ΔE*_trans_ (kJ mol^−1^)^b^	NA	3.32	0.88	2.21
*ΔE*_fitting_ (kJ mol^−1^)^b^	NA	−58.48	−58.27	−61.95
*ΔE*_ads_ (kJ mol^−1^)^b^	NA	−55.16	−57.39	−59.74
*ΔH* (kJ mol^−1^)^c^	NA	−62.6	−66.1	−66.9

NA: not applicable.

^a^Measured by Zn· · ·Zn atom-to-atom separations for unambiguous comparison.

^b^Obtained by GCMC-PDFT calculation.

^c^Obtained by fitting the van’t Hoff equation of GC retention factors measured at different temperatures.
