# Supplementary material for: Structural, energetic, and dynamic insights into the abnormal xylene separation behavior of hierarchical porous crystal
Source: Sci Rep. 2015 Jun 26;5:11537. doi: 10.1038/srep11537 (PMC4481377; doi:10.1038/srep11537)
Supplement: Supplementary Information [file srep11537-s1.pdf]

Supporting Information

Structural, energetic, and dynamic insights into the abnormal xylene separation behavior of hierarchical porous crystal

Jiao-Min Lin, Chun-Ting He, Pei-Qin Liao, Rui-Biao Lin, and Jie-Peng Zhang*

MOE Key Laboratory of Bioinorganic and Synthetic Chemistry, School of Chemistry and Chemical Engineering, Sun Yat-Sen University, Guangzhou 510275, China.

* E-mail: zhangjp7@mail.sysu.edu.cn

Additional experimental details.

Table S1. Crystallographic Data and Structural Refinements.

Table S2. Summary of the separation results for disubstituted benzene derivatives on the capillary column coated by **1**.

Figure S1. Coordination environments of **1**.

Figure S2. Network topology of **1**.

Figure S3. PXRD patterns of **1·g**.

Figure S4. Thermogravimetry curve of **1·g**.

Figure S5. Variable temperature PXRD patterns for **1·g**.

Figure S6. N_2 and CO_2 sorption isotherms for **1**.

Figure S7. SEM images of **1** coated capillary column.

Figure S8. Effect of temperature on the GC resolution.

Figure S9. Comparison of the sizes and shapes of xylene isomers.

Figure S10. Chromatograms on HP-5MS capillary column for GC separation of disubstituted benzene derivatives.

Figure S11. Effect of temperature on the GC selectivity.

Figure S12. van't Hoff plots for GC capacity factor k' and temperature T.

Figure S13. Perspective views of the quadrilateral aperture.

Figure S14. The preferential adsorption sites for oX, mX, and pX.

Figure S15. Close host-guest interactions in **1·oX**, **1·mX**, and **1·pX**.

Figure S16. Steric hindrance effects of hypothetical host-guest structures using mX to occupy the locations of pX and oX.

Additional experimental details

Fabrication of Capillary Column for GC Separation. A 25-mL round-bottom flask was charged with $Zn(CH_3COO)_2\cdot 2H_2O$ (0.022 g, 0.1 mmol), H_3pidba (0.039 g, 0.1 mmol) and a magnetic stirring bar. 8 mL of DMF and 1 mL of MeOH were added to the round-bottom flask and the mixture was stirred and heated at 105 ^{o}C for 2 days under air atmosphere. Submicrometer-scale crystals were collected by centrifugation of the suspension on the upper layer, and then washed with fresh ethanol for three times, which were used for fabrication of the GC capillary column.

The fused silica capillary (l = 16 m, d = 0.53 mm) was washed sequentially by NaOH (2 mol/L) for three times (immersed 3 h at the last time), ultrapure water (until the pH of outflow reached 7.0), HCl (1 mol/L) for three times (immersed 1 h at the last time), ultrapure water (until the pH of outflow reached 7.0), and methanol for three times, and then dried by a N_2 purge at 120 ^{o}C for 3 h. An ethanol (1 mL) suspension of **1** (25 mg) was pushed through the capillary column by N_2 flow with an inlet pressure of 0.4 MPa. The outflow (the ethanol suspension) was dried and estimated to be 16 mg, which indicated that about 9 mg of **1** was coated on the capillary. After coating, the capillary column was settled for 1 h for conditioning under N_2 flow. Further conditioning of the capillary column was carried out using a temperature program in a GC instrument: 5 °C min^{-1} from room temperature to 70 °C and stand for 30 min, then 5 °C min^{-1} to 150 °C and stand for 60 min, and then 5 °C min^{-1} to 250 °C and stand for 60 min. The temperature program was repeated for 3 times.

Calculation of Selectivity and Resolution. The selectivity factors ($\alpha_{B/A}$) for analytes A and B on the capillary column were calculated from gas chromatogram according to

$$\alpha_{B/A} = \frac{t_B - t_0}{t_A - t_0}$$

Where t_A, t_B and t_0 are the retention time of analytes A, B, and reference methanol, respectively, under the same operation conditions.

The resolution (R) for analytes A and B on the capillary column were calculated according to

$$R = \frac{t_B - t_A}{1/2(w_B + w_A)}$$

Where w_A and w_B are the peak width of analytes A and B, respectively.

Calculation of Thermodynamic Parameters. The enthalpy change (ΔH) and entropy change (ΔS) for the transfer of solutes from the mobile phase to the stationary phase were calculated from the van't Hoff equation.

$$\ln k' = \frac{-\Delta H}{RT} + \frac{\Delta S}{R} + \ln \Phi$$

where k' is the retention factor, R is the gas constant, T is the absolute temperature, and Φ is the phase ratio. Φ was defined as the volume of the stationary phase divided by the volume of the mobile phase.

Retention factor k' was calculated by

$$k' = \frac{t - t_0}{t_0}$$

where t is the retention time for the analyte and t_0 is the column void time under constant temperature gas chromatographic separation.

Φ was calculated by

$$\Phi = \frac{v_s}{v_0}$$

where V_S is the volume of the stationary phase in the column, and V_0 is the void volume of the column.

In this work, V_0 is 3.52 cm^3 (= 3.14 × (0.053/2 cm)^2 × 1600 cm), and V_S is 9.55 × 10^{-3} cm^3 (= 9.0 × 10^{-3} g/0.943 g cm^{-3}). Thus, $\ln \Phi$ was estimated to be −5.91.

Table S1. Crystallographic Data and Structural Refinements.

Complex	**1·g**	**1**	**1·ompX**
Formula	$C_{22}H_{13}N_3O_4Zn$	$C_{22}H_{13}N_3O_4Zn$	$C_{34}H_{28}N_3O_4Zn$
Formula weight	448.74	448.72	607.96
Temperature (K)	103(2)	103(2)	103(2)
Crystal system	Trigonal	Trigonal	Trigonal
Space group	*R*-3	*R*-3	*R*-3
a/Å	29.822(6)	30.298(2)	29.670(4)
c/Å	17.824(4)	17.881(2)	17.915(3)
V/Å^3	13728(7)	14215(2)	13658(3)
Z	18	18	18
D_c /g cm^{-3}	0.977	0.944	1.331
reflns coll.	48518	51572	47600
unique reflns	5559	5815	5552
R_{int}	0.0504	0.0618	0.0383
R_1 [$I > 2\sigma(I)$][a]	0.0563	0.0389	0.0574
wR_2 [$I > 2\sigma(I)$][b]	0.1890	0.1103	0.1823
R_1 (all data)	0.0576	0.0455	0.0579
wR_2 (all data)	0.1909	0.1407	0.1830
GOF	1.029	1.023	1.035

Table S1. (continued). Crystallographic Data and Structural Refinements.

Complex	**1·oX**	**1·mX**	**1·pX**
Formula	$C_{34}H_{28}N_3O_4Zn$	$C_{34}H_{28}N_3O_4Zn$	$C_{36.64}H_{31.30}N_3O_4Zn$
Formula weight	607.98	554.90	643.02
Temperature (K)	103(2)	103(2)	103(2)
Crystal system	Trigonal	Trigonal	Trigonal
Space group	*R*-3	*R*-3	*R*-3
a/Å	29.875(4)	29.608(4)	30.516(4)
c/Å	18.072(4)	18.158(3)	17.704(4)
V/Å^3	13968(4)	13785(3)	14278(4)
Z	18	18	18
D_c /g cm^{-3}	1.301	1.203	1.346
reflns coll.	49677	46705	48803
unique reflns	5668	5595	5808
R_{int}	0.0466	0.0626	0.0325
R_1 [$I > 2\sigma(I)$][a]	0.0668	0.0809	0.0356
wR_2 [$I > 2\sigma(I)$][b]	0.2009	0.2605	0.0981
R_1 (all data)	0.0814	0.0843	0.0366
wR_2 (all data)	0.2379	0.2666	0.0993
GOF	1.014	1.040	1.046

$^aR_1 = \Sigma||F_o| - |F_c||/\Sigma|F_o|$, $^bwR_2 = [\Sigma w(F_o^2 - F_c^2)^2/\Sigma w(F_o^2)^2]^{1/2}$.

Table S2. Summary of the separation results for disubstituted benzene derivatives on the capillary column coated by **1**.

Analyte		Boiling point (°C)	Retention time (min)	RSD (%) (n = 5)			ΔH (kJ mol^{-1})	ΔS (kJ mol^{-1})	R^2
				Retention time	Half peak width	Peak area			
xylene	*o*-	144.4	2.42	0.04	1.24	0.85	−62.6 ± 0.3	−67.9 ± 0.7	0.99991
	m-	139.1	2.89	0.06	1.72	1.60	−66.1 ± 0.3	−73.5 ± 0.7	0.99989
	p-	138.4	3.34	0.02	1.10	2.37	−66.9 ± 0.2	−73.3 ± 0.6	0.99993
ethyltoluene	*o*-	165.2	1.11	0.37	0.93	1.39	−67.6 ± 0.2	−72.6 ± 0.4	0.99997
	m-	161.3	1.45	0.12	1.32	1.72	−71.8 ± 0.1	−78.3 ± 0.3	0.99998
	p-	162	1.85	0.19	1.03	1.14	−73.1 ± 0.2	−78.6 ± 0.4	0.99998
chlorotoluene	*o*-	159.3	1.36	0.39	2.0	2.82	−68.6 ± 0.9	−77.3 ± 1.9	0.99929
	m-	161.6	1.75	0.29	2.35	3.35	−71.6 ± 0.3	−80.8 ± 0.7	0.99991
	p-	162.0	2.36	0.27	3.32	4.17	−73.4 ± 0.6	−81.5 ± 1.3	0.99971
methylanisole	*o*-	171.8	1.20	0.21	1.18	2.56	−67.3 ± 0.3	−71.7 ± 0.7	0.9999
	m-	176.5	1.57	0.17	1.64	1.88	−69.0 ± 0.3	−72.4 ± 0.7	0.9999
	p-	176.7	1.96	0.17	2.59	0.42	−70.8 ± 0.2	−73.6 ± 0.4	0.99997

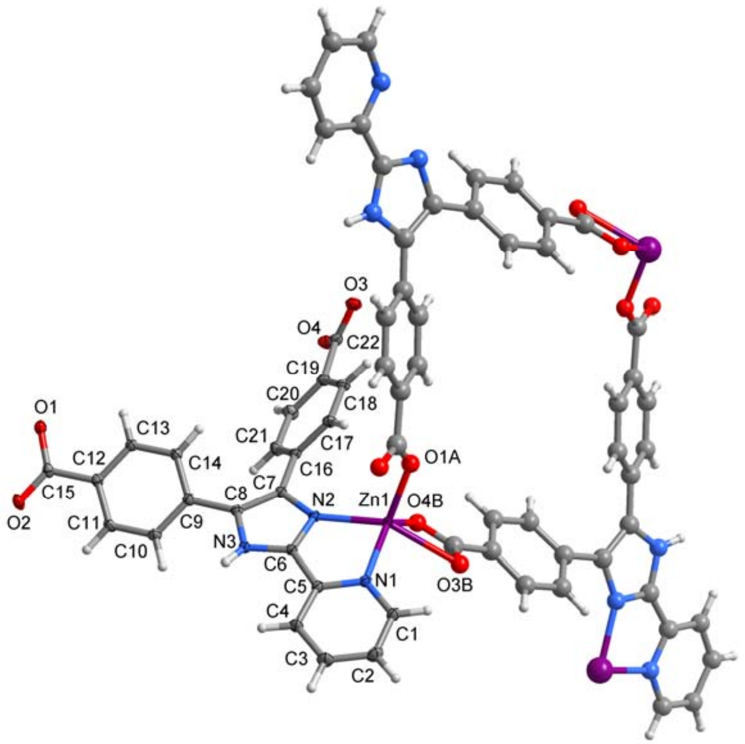

Figure S1. (a) Perspective view of the coordination environments of **1** (probability ellipsoids drawn at 30% for the asymmetric unit). Symmetry codes: A = 2/3−*x*+*y*, 4/3−*x*, 1/3+*z*; B = 2/3+*x*−*y*, 1/3+*x*, 4/3−*z*.

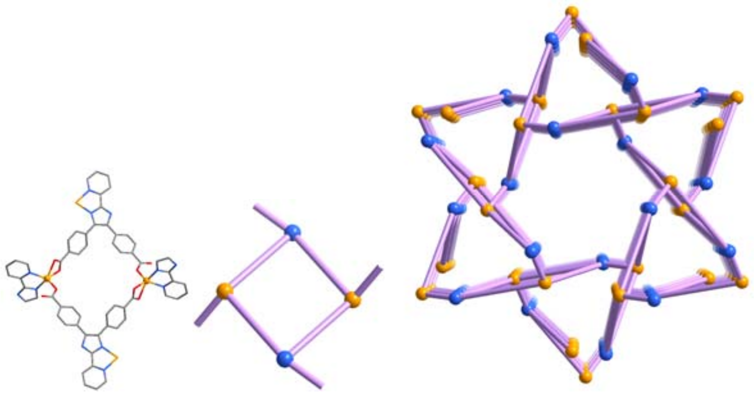

Figure S2. Definitions of the 3-connected nodes (yellow sphere, Zn(II) ion; blue sphere, $Hpidab^{2-}$ ligands) and the **nbo-a** network.

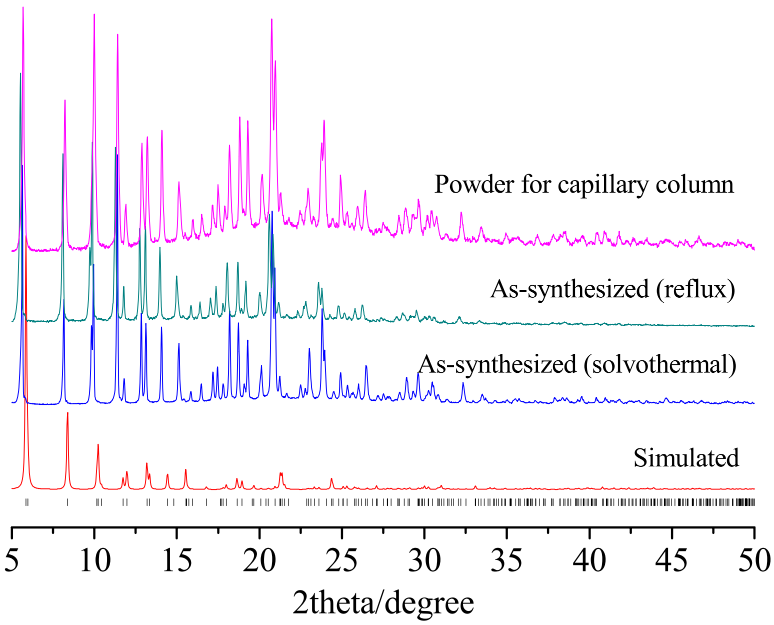

Figure S3. PXRD patterns of **1·g**.

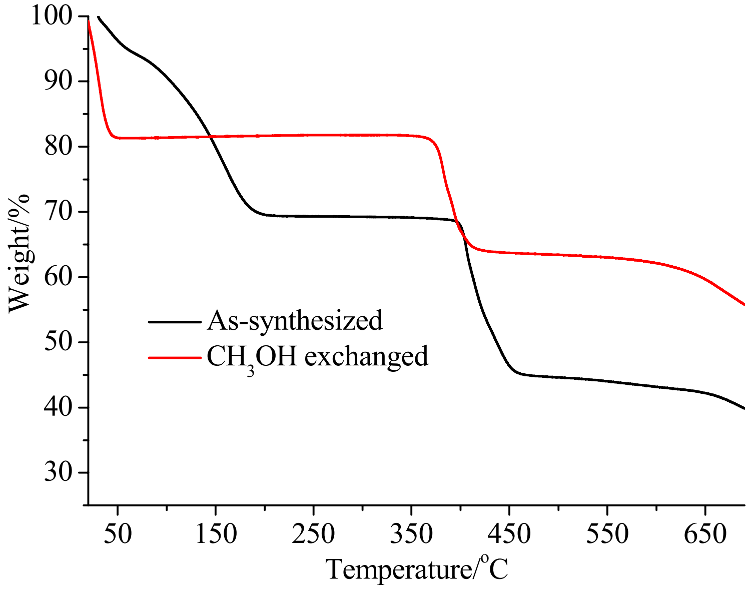

Figure S4. Thermogravimetry curve of as-synthesized and CH_3OH exchanged **1·g**.

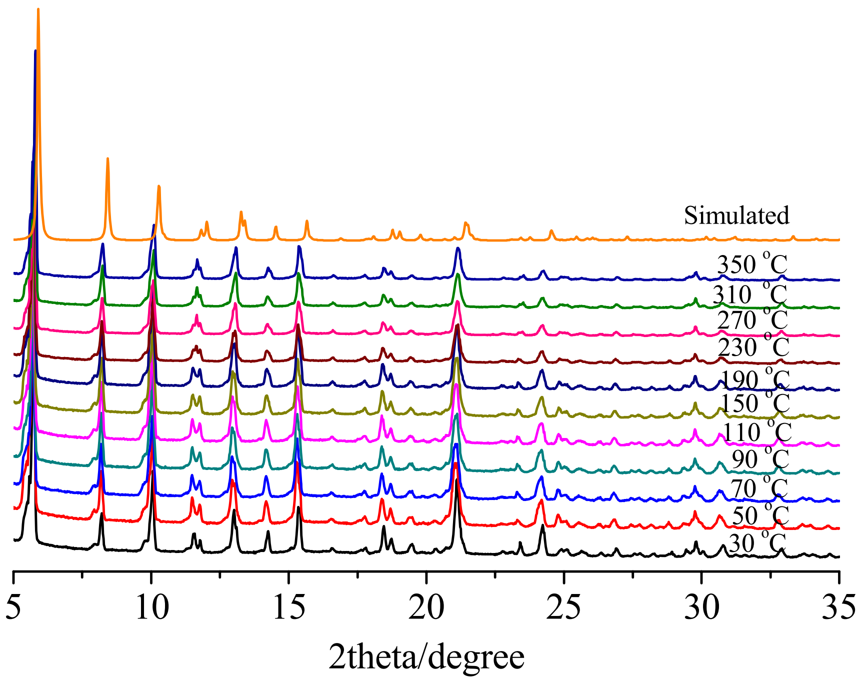

Figure S5. Variable temperature PXRD patterns under N_2 for CH_3OH exchanged **1·g**.

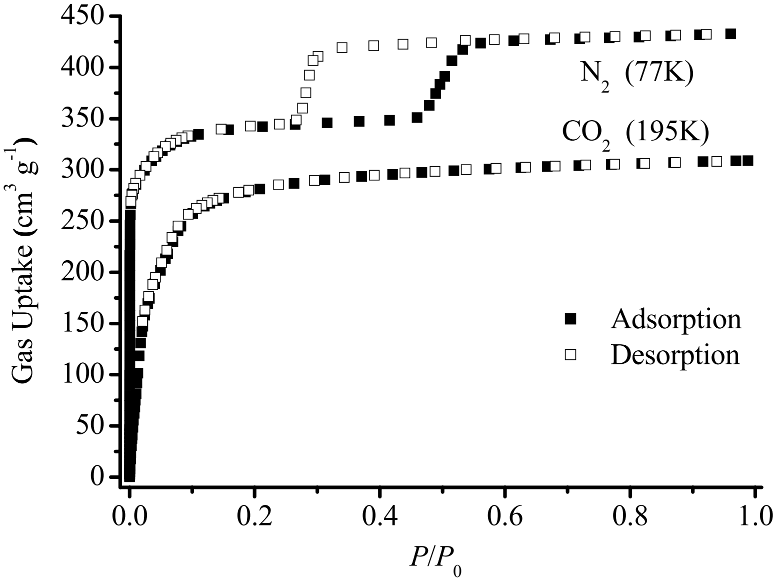

Figure S6. N_2 and CO_2 sorption isotherms for **1**.

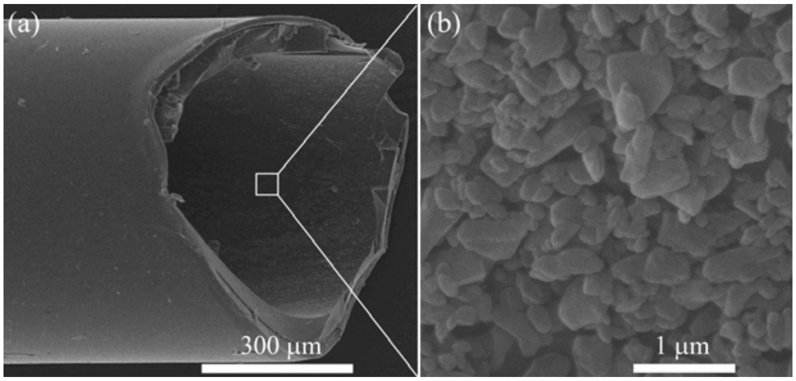

Figure S7. SEM images of (a) the cross section of **1** coated capillary column; and (b) **1** deposited on the inner wall of the capillary column.

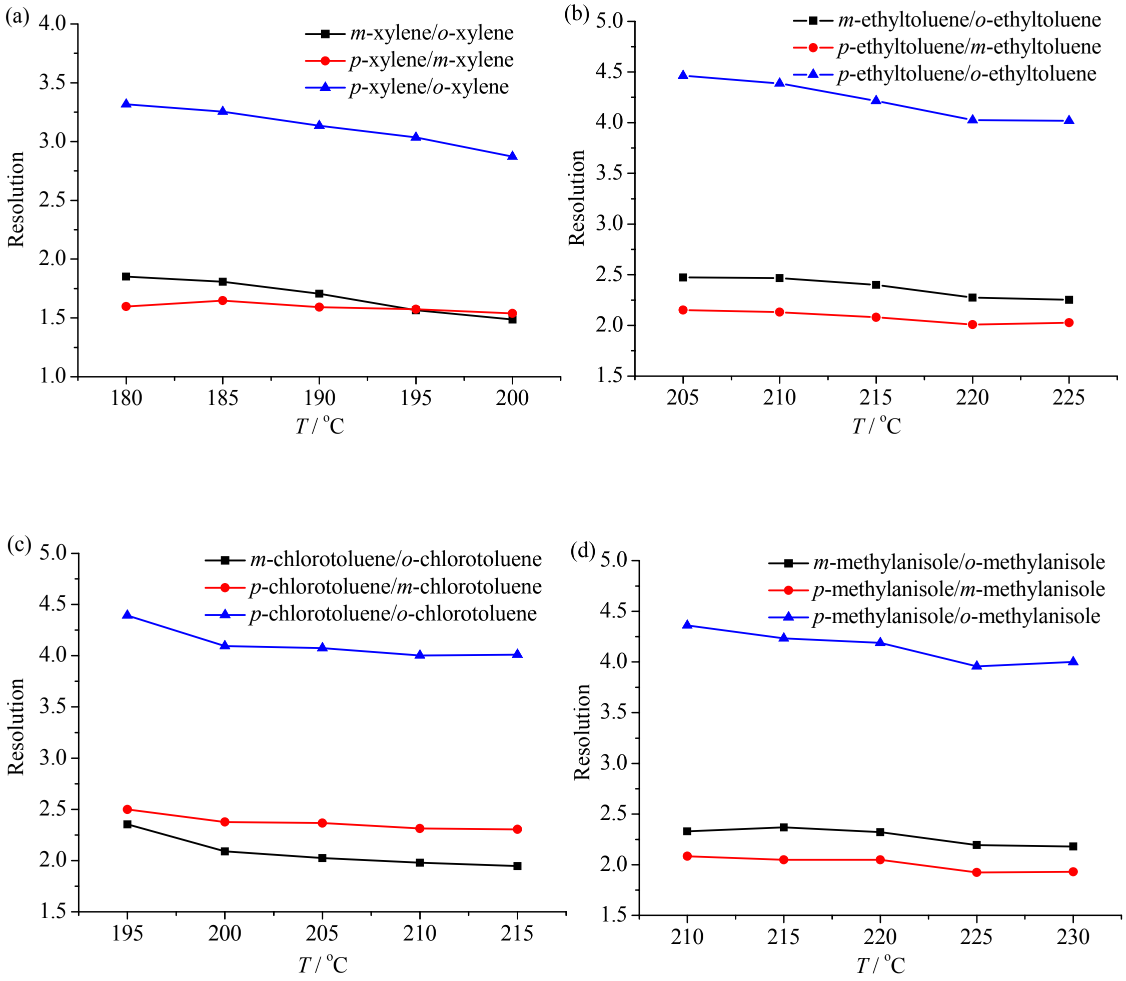

Figure S8. Effect of temperature on the GC resolution of (a) xylene, (b) ethyltoluene, (c) chlorotoluene, and (d) methylanisole isomers.

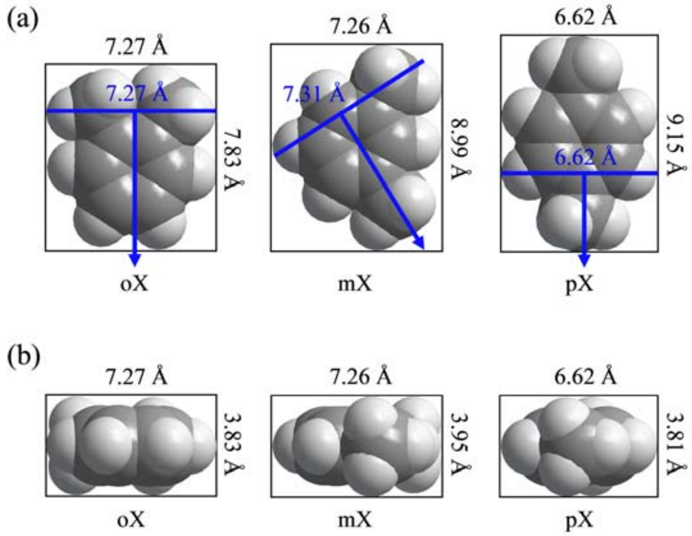

Figure S9. Comparison of the sizes and shapes of xylene isomers. Perspective views perpendicular to the (a) largest (width × length) and (b) smallest (thickness × width) cross sections. The black rectangles represent the conventional description of their molecular sizes. The blue arrows represent the most possible directions of the molecules inserting into a small aperture. Each blue line perpendicular with a blue arrow represents the widest section of the molecule, which gives rise to the largest host-guest attractive interaction or steric hindrance.

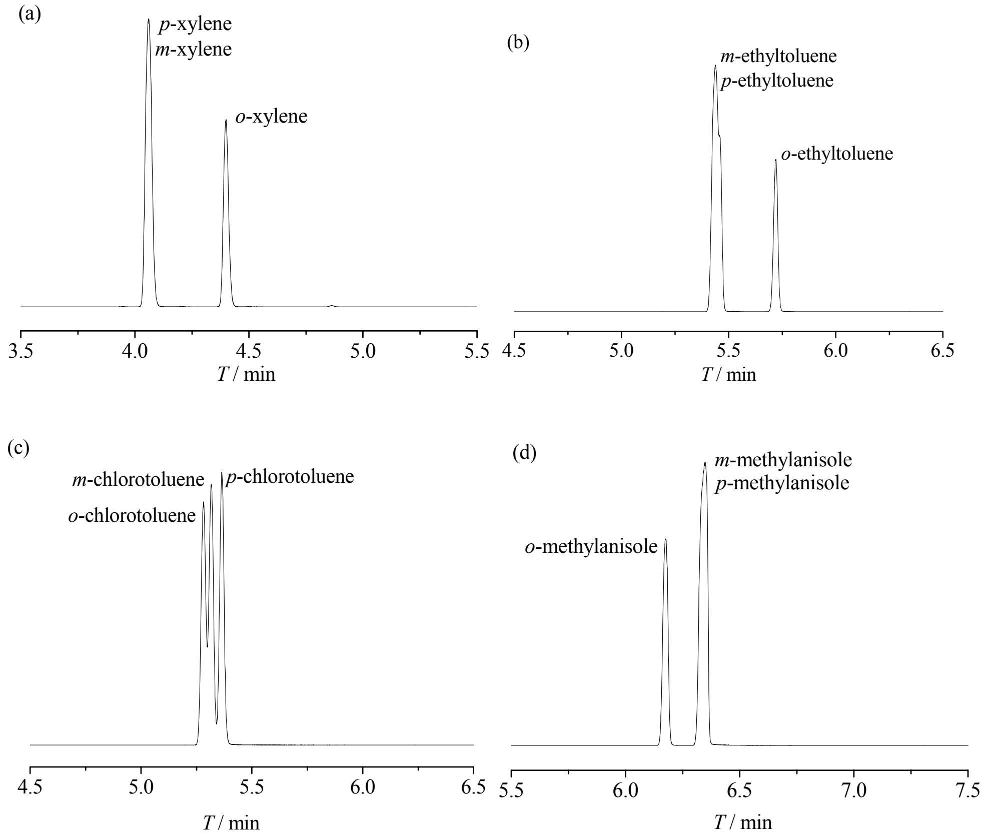

Figure S10. Chromatograms on commercial HP-5MS capillary column (30 m long × 0.25 mm i.d.) for GC separation of (a) xylene, (b) ethyltoluene, (c) chlorotoluene, and (d) methylanisole isomers using a temperature program of 50 to 200 ^{o}C with a rate of 10 °C min^{-1}, under a He flow rate of 1.2 mL min^{-1} and a split ratio of 15:1. The experiments were performed on an Agilent 5975C system equipped with a mass spectrometry detector (very small amounts of analytes, i.e., 0.0067 μg for each isomer, were injected, so that the high-sensitivity detector was used).

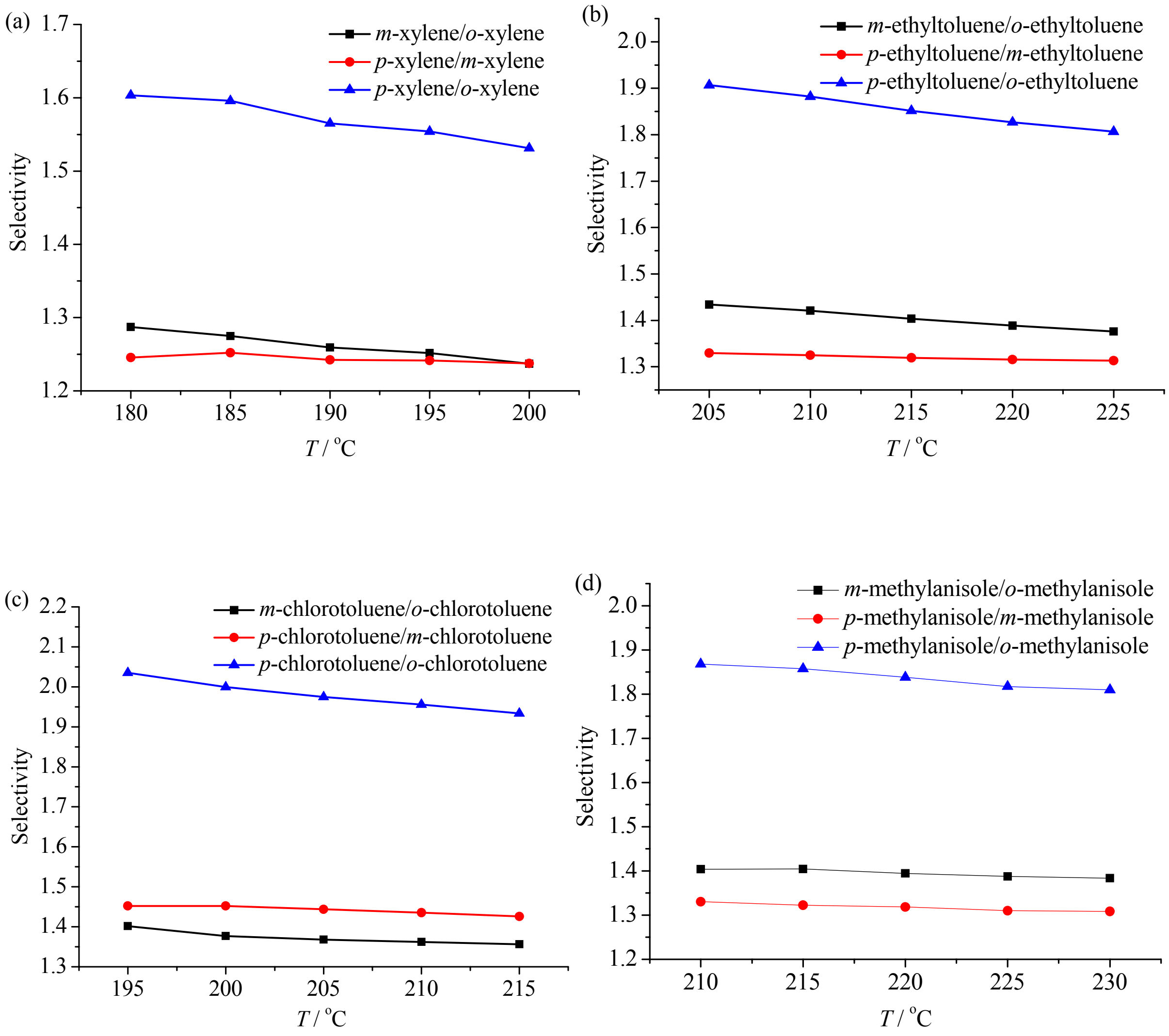

Figure S11. Effect of temperature on the GC selectivity of (a) xylene, (b) ethyltoluene, (c) chlorotoluene, and (d) methylaniosle isomers.

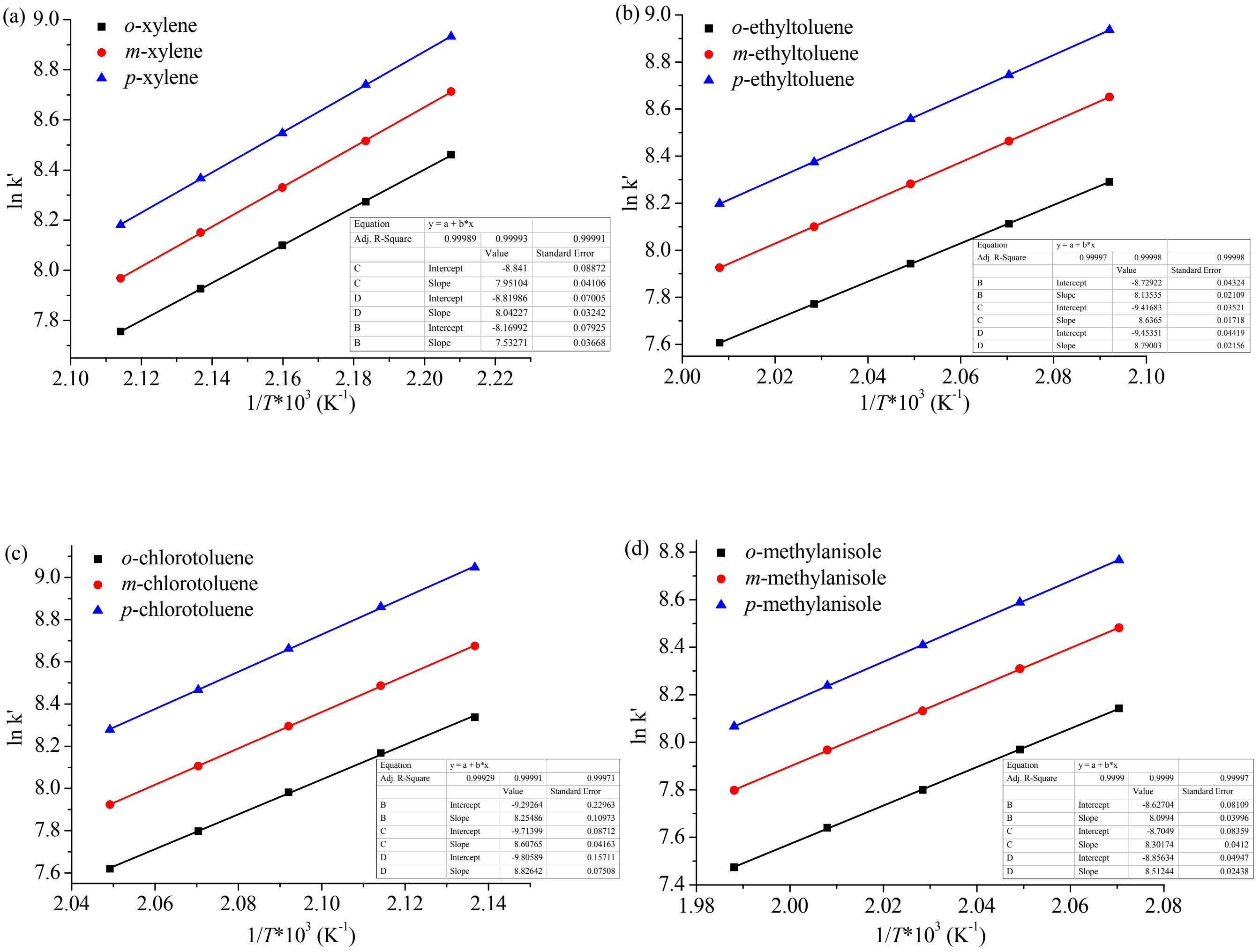

Figure S12. van't Hoff plots for the GC capacity factor k' and temperature T.

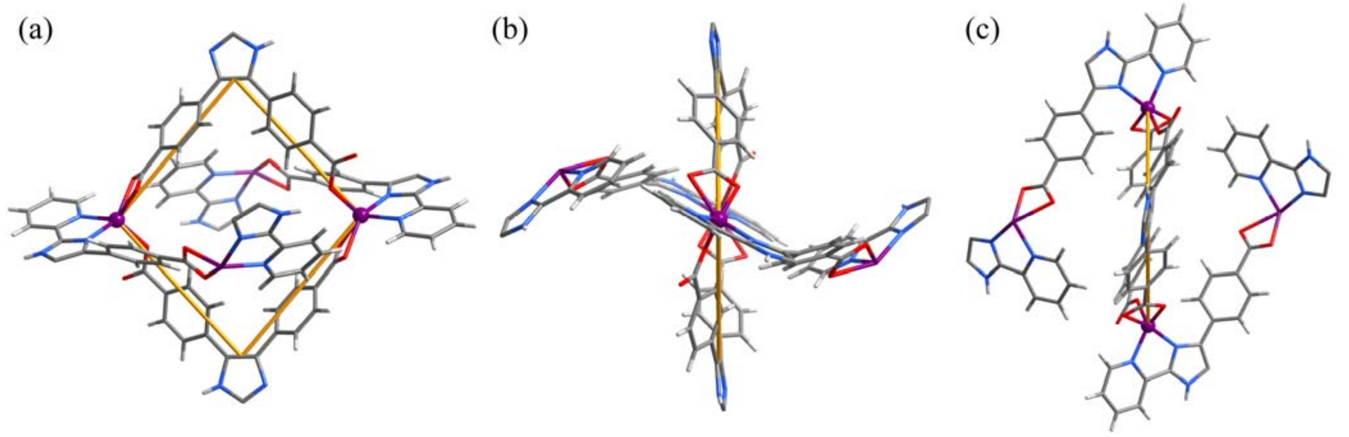

Figure S13. Perspective views of the quadrilateral aperture (a) perpendicular to aperture plane, (b) parallel to the Zn···Zn diagonal, and (c) parallel to the imidazole···imidazole diagonal.

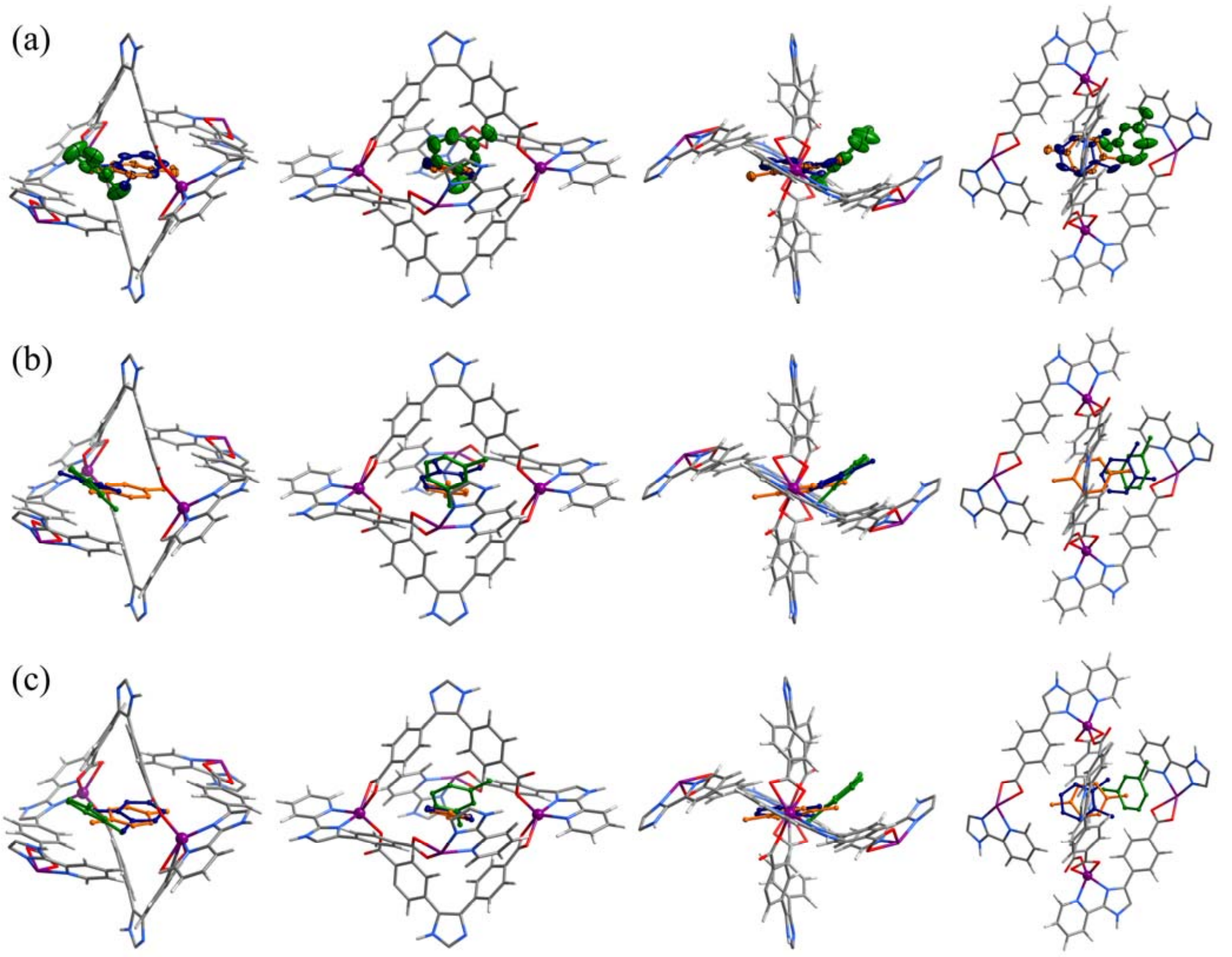

Figure S14. Comparison of positions of guest molecules (oX, dark blue; mX, green; pX orange; hydrogen atoms are omitted for clarity) at the primary adsorption site (i.e. the small aperture of **1**, drawn in multi-color stick mode; Zn purple, C dark gray, H light gray, N blue, O red) observed by (a) single-crystal X-ray diffraction (thermal ellipsoids drawn at 30% probability), (b) GCMC simulation and (c) GCMC-PDFT calculation. From left to right, the structures are projected along a common direction (same as that for Figure 1), perpendicular to the aperture plane, parallel to the Zn···Zn diagonal, and parallel to the imidazole···imidazole diagonal, respectively.

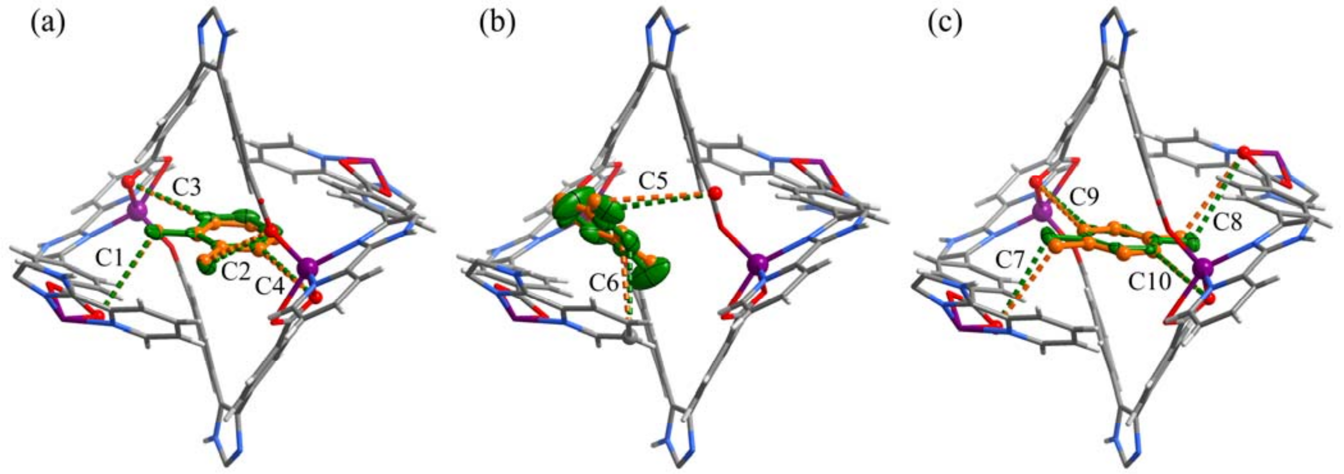

Figure S15. Close host-guest interactions in (a) **1·oX**, (b) **1·mX**, and (c) **1·pX**. Close contacts revealed by single-crystal X-ray diffraction (green) and GCMC-PDFT calculation (orange) are 3.798(26) and 4.076 Å for C1, 3.396(14) and 3.428 Å for C2, 3.774(8) and 3.895 Å for C3, 3.807(8) and 3.683 Å for C4, 3.960(15) and 3.679 Å for C5, 3.556(9) and 3.689 for C6, 3.837(4) and 3.599 Å for C7, 3.837(4) and 3.774 Å for C8, 3.607(3) and 3.839 Å for C9, 3.607(3) and 3.694 Å for C10, respectively.

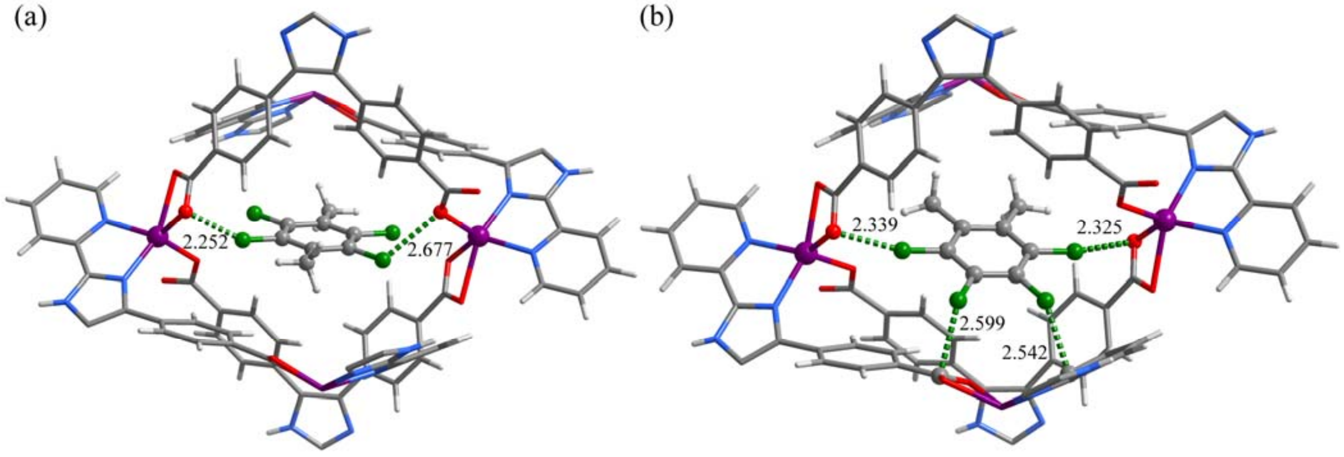

Figure S16. Steric hindrance effects of hypothetical host-guest structures using mX to occupy the locations of (a) pX and (b) oX. Green spheres represent all possible positions for location of the methyl groups (hydrogen atoms are omitted for clarity) of mX molecules, whose phenyl rings adopt the same position of pX/oX (stick mode in gray), and green dashed sticks represent the separations between the host framework atom and the methyl carbon atom of mX, which are much shorter than acceptable values.
